# Desmoplastic Small Round Cell Tumor Presenting as an Intra/Extracranial Mass

**DOI:** 10.7759/cureus.55494

**Published:** 2024-03-04

**Authors:** Meshari Almutairi, Khalid T Alghamdi, Othman T Almutairi, Salman T Almalki, Abdulrahman Y Alturki

**Affiliations:** 1 Department of Neurosurgery, Prince Mohammed Bin Abdulaziz Hospital, Riyadh, SAU; 2 Department of Neurosurgery, King Faisal Specialist Hospital & Research Centre, Riyadh, SAU; 3 Department of Adult Neurosurgery, King Fahad Medical City, Riyadh, SAU; 4 Department of Pathology, King Fahad Medical City, Riyadh, SAU

**Keywords:** skull lesion, brain metastasis, intra/extracranial mass, dsrct, desmoplastic small round cell tumor

## Abstract

Desmoplastic small round cell tumors (DSRCTs) are highly malignant tumors, with distinct reciprocal chromosome translocation (11;22)(p13;q12). Intracranial metastasis is a very rare complication of this tumor, with only a few cases reported in the literature. To our knowledge, this is the only case presenting an extracranial extension of intracranial metastasis of DSRCT. A 33-year-old man was diagnosed with DSRCT in the pelvic cavity. He presented with a scalp lump and right-sided weakness. A biopsy showed metastasis from DSRCT. Metastatic DSRCT to the brain is extremely rare. Surgical resection followed by adjuvant treatment, including chemotherapy and radiation, is indicated as it has a poor prognosis. Moreover, aggressive treatment is warranted to prevent progression and relapse.

## Introduction

Desmoplastic small round cell tumor (DSRCT) is a malignant mesenchymal neoplasm that usually occurs in the abdomen [1]. It is known to be a male predominant disease, with incidence reaching approximately 90% [2]. This neoplasm has a fusion of the EWSR1-WT1 gene, and it shows a polyphenotypic immunoprofile with co-expression of multiple markers [1,3-5]. It was first described by Gerald and Rosai in 1989, who proposed that it arose from progenitor cells during the development stage [1]. It is a highly malignant small cell tumor with distinct reciprocal chromosome translocation t(11;22)(p13;q12) [6]. The clinic presentation includes abdominal pain, distention, or bowel obstruction which can be noticed as vomiting or constipation. Microscopically, it appears as a nest of small blue cells interposed in the desmoplastic stroma with multiple positive markers such as epithelial (cytokeratins and epithelial membrane antigen), myogenic (desmin), mesenchymal (vimentin), and neural (neuron-specific enolase and CD56) [7]. DSRCT mainly affects young adult males with a predilection to involve intra-abdominal organs and the peritoneum. Intracranial metastasis is very rare with a few case reports [3]. Here, we present a case of intracranial metastatic DSRCT with extension into the skull and subcutaneous tissue presenting uniquely as a scalp lump.

## Case presentation

We present the case of a 33-year-old male with a diagnosis of pelvic desmoplastic round cell tumor in Jordan. The patient received neoadjuvant chemotherapy with etoposide and ifosfamide (vinCRIStine, DOXOrubicin, and cyclophosphamide (IE-VAC) therapy) for four cycles. Subsequently, he underwent resection with a positive margin. His chemotherapy was then changed to cyclophosphamide and topotecan as he had a poor response to neoadjuvant chemotherapy. The patient was transferred to Saudi Arabia to continue his treatment. He received seven cycles of cyclophosphamide and topotecan, with the last two cycles without cyclophosphamide due to the start of radiation therapy. The patient was started on external-beam radiation (40 Gy/30 fractions) with topotecan completed.

The next year, the patient developed disease recurrence with peritoneal metastasis. A peritonectomy with hyperthermic intraperitoneal chemotherapy was done. No residual disease was identified intraoperatively. After the surgery, he received temozalomide and irinotecan on days 1-5 and 8-12 and had completed six cycles six months later. Following this, he was kept on active surveillance.

Eleven months later, he presented to emergency with right-sided weakness due to left frontal metastasis in addition to the progression of the mediastinal mass, peritoneal deposits, and abdominal lymph nodes. There were also early bony lesions at the left humerus and right trochanter.

**Figure 1:**
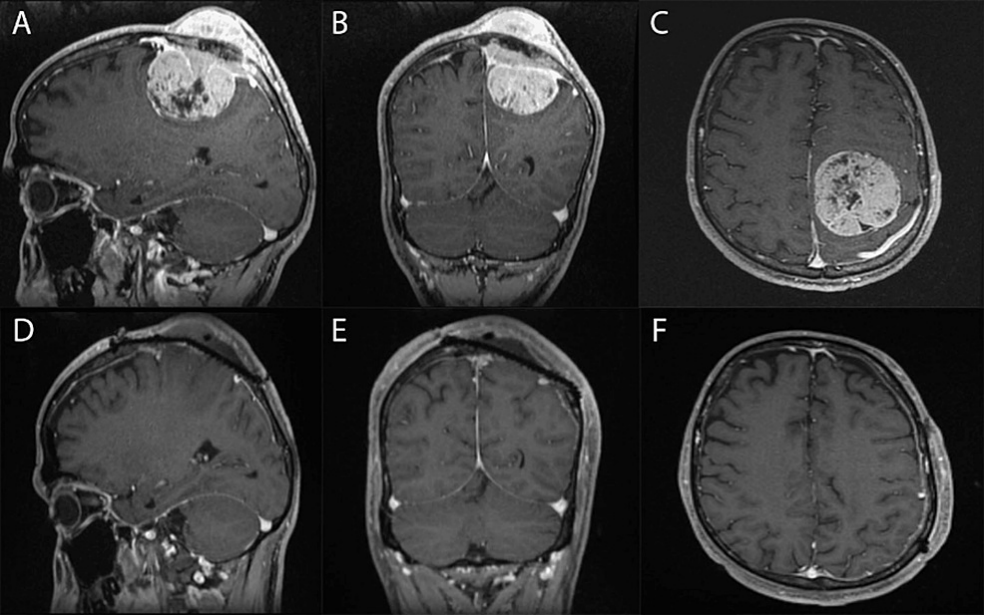
Pre and postoperative magnetic resonance imaging of the brain. A, B, and C: Preoperative sagittal, coronal, and axial magnetic resonance imaging (MRI) of the brain (T1 with contrast) showing a large parasagittal intra and extra-axial enchaining brain lesion in the left frontal lobe with an invasion of the superior sagittal sinus, skull, and scalp. D, E, and F: Postoperative sagittal, coronal, and axial MRI of the brain (T1 with contrast) showing gross total resection of the lesion with minimal residual over the superior sagittal sinus and skull defect covered by titanium mesh cranioplasty.

**Figure 2:**
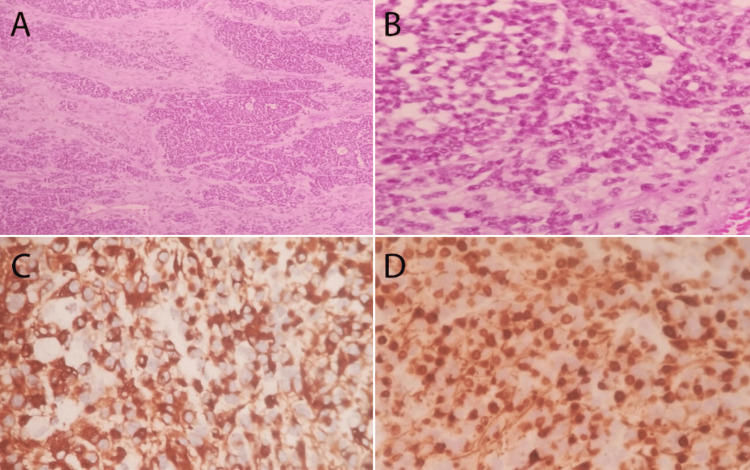
Pathology slides. A and B: Small round cell tumor composed of small round blue cells in desmoplastic stroma (hematoxylin and eosin: ×10, ×40). C: Desmin-positive tumor cells with a peculiar dot-like pattern. D: Nuclear staining of WT1 in tumor cells.

The patient underwent craniotomy with maximum safe resection of the left frontoparietal metastatic mass (Figure 1). Subsequently, he completed brain radiation (20 Gy/S fractions) and eight cycles of dactinomycin as chemotherapy. Postoperative pathology showed a small round cell tumor with small round blue cells in the desmoplastic stroma (Figure 2). After one year of follow-up, he reported mild residual weakness improving with physiotherapy. The Eastern Cooperative Oncology Group scale score was 1.

## Discussion

DSRCT is a rare type of small cell (blue) tumor [8]. Very few intracranial primary/metastatic cases have been reported in the literature, with only 10 primary cases and one metastatic brain tumor reported (Table 1) [8-13]. Our case is unique because it presented as a scalp mass. Investigation showed a large parasagittal intra/extracranial mass invading the superior sagittal sinus and the skull. To our knowledge, no similar presentation has been reported in the literature.

**Table 1:** A literature review of previous cases of DSRCT with intra/extracranial metastasis. DSRCT = desmoplastic small round cell tumor; NSE = neuron-specific enolase; PCR = polymerase chain reaction; CPA = cerebellopontine angle

N	Author	Age/Sex	Presentation	Location	Histopathology and immunohistochemistry	Cytogenetics	Treatment
1	Tison et al., 1996 [9]	24 years/Male	Headache, vomiting, vertigo, and impaired hearing	Posterior fossa lesion	Small, round tumor cells of primitive appearance growing as well-defined nests separated by abundant desmoplastic stroma. Positive for keratin, desmin, and NSE	EWS-WT1 fusion gene by PCR	Partial resection of the tumor. Chemotherapy (PCNU, V16, cisplatin, and intracranial MTX). Radiotherapy
2	Bouchireb et al., 2008 [10]	6 years/Female	Headache and complex partial seizures	Right temporal lobe lesion	Small malignant cells with hyperchromatic nuclei and eosinophilic cytoplasm embedded in a fibromyxoid stroma. Positive vimentin, desmin, and synaptophysin	EWS-WT1 translocation by multiplex RT-PCR	Complete excision of the tumor. Chemotherapy P6 protocol (CAV, ifosfamide, and etoposide) Focal conformal irradiation to the tumor bed at 54 Gy
3	Neder et al., 2009 [11]	37 years /Male	Left-sided hearing loss and tinnitus. Progressive left leg weakness 6 months later	Left cerebellopontine angle. Spinal drop metastasis	Sheets of small- to medium-sized cells with high nuclear-cytoplasmic ratios and round-to-oval hyperchromatic nuclei with inconspicuous nucleoli in addition to a desmoplastic stroma. Positive staining for EMA, CAM 5.2, desmin, and nuclear INI-1	fusions of EWS–WT1 genes by PCR	Subtotal resection of the CPA lesion, followed by stereotactic irradiation to the tumor bed. Debulking of spinal intradural tumor nodules. Chemotherapy (carboplatinum, temozolomide). Whole brain and spine radiation
4		39 years /Male	Gait imbalance, bilateral lower limb weakness, and urinary and fecal incontinence	Spinal leptomeningeal metastasis/left cerebellum	Oval to irregular nuclei with coarse chromatin and scant cytoplasm. Positive staining for EA, CAM 5.2, desmin, and nuclear INI-1	Fusions of EWS–WT1 genes by PCR	Decompressive T12–L5 laminectomy. Chemotherapy (cisplatin, etoposide, and Holoxan). Radiotherapy
5	Thondam et al., 2015 [12]	27 years /Male	Excessive tiredness, lethargy, and loss of libido. Headaches, drowsiness, and bitemporal hemianopia one year later	Suprasellar mass	Nests and cords of fairly uniform tumor cells characterized by hyperchromatic nuclei and indistinct cytoplasm distributed in a desmoplastic stroma. Positive staining for CAM 5.2, vimentin, and desmin	EWS-WT1 translocation by RT-PCR	Near-total resection. Fractionated conformal radiotherapy but discontinued due to poor general condition
6	Umeda et al., 2015 [13]	16 years /Male	Severe headache	Metastatic cerebellar/pineal body/skull lesions	No pathology for head lesions. Primary lesion: Groups of small, round, undifferentiated cells embedded in a desmoplastic stroma. Positive for NSE, desmin, cytokeratin, and WT1	EWS-WT1 gene fusion detected by RT-PCR	Open biopsy of lymph nodes. Chemotherapy: A modified protocol of the P6 regimen. Gross total resection of the primary tumor in the pelvic floor. Whole abdominopelvic radiation therapy. Irinotecan and temozolomide for metastatic brain lesions
7	Lee et al., 2020 [8]	13 years /Male	Seizures	Right temporal	Small round cells embedded in a desmoplastic stroma. Positive staining for desmin, EMA, synaptophysin, and NeuN	EWSR1-WT1 gene fusion by targeted next-generation DNA sequencing	Gross total resection. No adjuvant chemotherapy. Six weeks of radiation therapy
8		6 years /Male	-	Left occipital	Solid sheets of tumor cells with hyperchromatic nuclei, only focal desmoplasia, a high mitotic index, and a malignant appearance. Positive staining for desmin	EWSR1-WT1 gene fusion by targeted next-generation DNA sequencing	-
9		25 years /Male	Left-hand numbness and headache	Left cerebellum	The tumor cells had large pleomorphic nuclei with abundant mitoses. No desmoplastic stroma. Positive staining for desmin, EMA, CAM 5.2, synaptophysin, and NeuN	EWSR1-WT1 gene fusion by targeted next-generation DNA sequencing	Gross total resection. No adjuvant therapy
10		11 years /Male	Progressive right-sided weakness	Left parietal	Markedly desmoplastic stroma. Positive staining for desmin, EMA cytokeratin, synaptophysin, and NeuN	EWSR1-WT1 gene fusion by targeted next-generation DNA sequencing	Gross total resection. Chemotherapy (six cycles of vincristine and cyclophosphamide). Radiotherapy 55 Gy
11		8 years /Male	Headaches	Right frontal	Only focal desmoplastic stroma. Positive staining for desmin, SMA, and synaptophysin	EWSR1-WT1 fusion transcript by RT-PCR	Gross total resection. Chemotherapy (P6 protocol). Radiotherapy 55 Gy

Diagnosis of DSRCT was difficult in the past, but now because of advancements in genetic analysis, it has become easier. Histopathology classical shows a small round blue cell tumor with a desmoplastic stroma. Immunohistochemistry shows positivity for desmin, EMA, CAM 5.2, cytokeratin, and synaptophysin. For definitive diagnosis, *EWSR1-WT1* gene fusion can be demonstrated by cytogenetic analysis.

Reported adjuvant treatment of such a rare tumor is the P6 protocol which consists of seven courses of chemotherapy, including high-dose cyclophosphamide, doxorubicin, vincristine, ifosfamide, and etoposide. The P6 protocol has been shown to prolong progression-free survival in patients with abdominopelvic DSRCT [1,7]. DSRCT is an aggressive tumor with poor survival, warranting chemotherapy and radiation therapy following surgical excision with close follow-up as a high rate of relapse has been reported [13].

## Conclusions

Metastatic brain DSRCT is rare with few cases reported in the literature. Surgical resection followed by adjuvant treatment, including chemotherapy and radiation, is indicated as it has a poor prognosis. Aggressive treatment and keeping patients on active surveillance is warranted to prevent progression and relapse.
